# Integrative Spatial Transcriptomics and Immunoinformatics for Prognostic Multi-Epitope Vaccine Construct Prediction Against Synovial Sarcoma

**DOI:** 10.3390/ph19020282

**Published:** 2026-02-07

**Authors:** Maha A. Aljumaa, Maher S. Alwethaynani, Hanan Abdulrahman Sagini, Fakhria A. Al-Joufi, Ghulam Nabi

**Affiliations:** 1 Department of Biology, College of Science, Princess Nourah Bint Abdulrahman University, P.O. Box 84428, Riyadh 11671, Saudi Arabia; maaljumaa@pnu.edu.sa; 2 Department of Clinical Laboratory Sciences, College of Applied Medical Sciences, Shaqra University, P.O. Box 84428, Alquwayiyah 11961, Saudi Arabia; malwethaynani@su.edu.sa; 3 Department of Biological Sciences, College of Science, University of Jeddah, P.O. Box 84428, Jeddah 21959, Saudi Arabia; hsajini@uj.edu.sa; 4 Department of Pharmacology, College of Pharmacy, Jouf University, P.O. Box 84428, Aljouf 72341, Saudi Arabia; faaljoufi@ju.edu.sa; 5 Department of Zoology, The University of Swat, P.O. Box 19120, Swat 19120, Pakistan

**Keywords:** synovial sarcoma, *FKBP10*, multi-epitope vaccine, molecular docking, molecular dynamics

## Abstract

**Background/Objectives:** Synovial sarcoma (SS) is a rare and aggressive soft-tissue malignancy characterized by complex molecular alterations and poor prognosis, highlighting the need for targeted immunotherapeutic strategies. This study aimed to design a rational multi-epitope vaccine targeting the *FKBP10* oncoprotein to elicit effective immune responses against SS. **Methods:** Transcriptomic data from the GEO dataset GSE144190, comprising 10 tumor and 9 normal tissue samples, were analyzed to identify differentially expressed genes (DEGs). **Results:** Our findings revealed significantly upregulated *FKBP10* with a log2 fold change of 3.55, baseMean expression of 1521.84, and adjusted *p*-value of 8.37 × 10^−26^. Mutational analysis across 7782 sarcoma samples indicated a low alteration frequency of ~1.5%, primarily missense variants. Functional mapping showed *FKBP10* as a hub interacting with multiple collagen chains and chaperone proteins, implicating its role in extracellular matrix organization and protein folding. Linear B-cell epitope prediction identified 17 epitopes (6–21 amino acids), while T-cell mapping yielded 10 MHC class I and 9 MHC class II high-affinity epitopes, all antigenic (VaxiJen > 0.5) and non-allergenic. A multi-epitope vaccine was constructed incorporating a 50S ribosomal protein L22 adjuvant, linkers, and a 6× histidine tag. Physicochemical analysis showed a molecular weight of 36.43 kDa, pI 6.97, instability index 31.79, aliphatic index 64.88, and GRAVY −0.509, indicating stability and hydrophilicity. Structural modeling validated 82.5% residues in favored regions. Molecular docking revealed strong binding with TLR4 (−9.7 kcal/mol) and TLR9 (−9.4 kcal/mol), and 200 ns molecular dynamics simulations confirmed stable RMSD trajectories, low RMSF at binding residues (<4 Å), persistent hydrogen bonding, compact radius of gyration (38–42 Å for TLR4; ~20 Å for TLR9), favorable total energy (−1400 to −1500 kcal/mol for TLR4; −650 to −720 kcal/mol for TLR9), and stable SASA (~52,000–54,000 Å^2^). **Conclusions:** These findings demonstrate that the *FKBP10* multi-epitope vaccine is structurally stable, immunogenic, and capable of engaging key innate immune receptors, supporting its potential as a promising immunotherapeutic candidate for synovial sarcoma.

## 1. Introduction

Synovial sarcoma (SS) is a rare and aggressive soft tissue malignancy, predominantly affecting adolescents and young adults and accounts for approximately 5–10% of all soft tissue sarcomas worldwide [1]. According to the World Health Organization (WHO), the global incidence of synovial sarcoma is estimated at 1–3 cases per million individuals annually, with mortality rates remaining high due to late diagnosis and metastatic progression [2]. The pathogenesis of SS is largely driven by characteristic chromosomal translocations, most notably t(X;18)(p11;q11), resulting in the formation of the SS18-SSX fusion oncoprotein, which dysregulates transcriptional and epigenetic programs. Other genetic aberrations, including mutations in TP53, CDKN2A, and BCL2, contribute to tumor proliferation, evasion of apoptosis, and enhanced metastatic potential. These molecular alterations underscore the aggressive nature of SS and the need for targeted interventions that can effectively modulate the underlying oncogenic mechanisms [3]. Current therapeutic strategies for SS primarily include surgical resection, often combined with adjuvant radiotherapy or chemotherapy. Wide local excision with negative margins remains the gold standard to minimize local recurrence, while conventional chemotherapeutic agents such as ifosfamide and doxorubicin are employed to manage metastatic or unresectable disease [4]. Despite these interventions, the overall survival rates for SS patients remain limited, with a 5-year survival of approximately 50–60% for localized tumors and substantially lower rates for metastatic cases. Radiotherapy, while beneficial for local control, carries the risk of long-term tissue damage, and chemotherapy often produces systemic toxicities with variable response rates. These limitations highlight the urgent need for innovative therapeutic modalities that can provide durable, tumor-specific efficacy with reduced adverse effects [5].

In recent years, cancer vaccines have emerged as a promising strategy for harnessing the host immune system to target tumor-specific antigens, offering a complementary or alternative approach to traditional therapies [6]. Unlike chemotherapy and radiotherapy, vaccines can induce long-lasting immunological memory and selectively target malignant cells while sparing healthy tissues [7]. Multi-epitope vaccines, in particular, allow the simultaneous presentation of multiple antigenic determinants to both B-cells and T-cells, enhancing the breadth and robustness of the immune response. In the context of SS, which is driven by well-characterized genetic elements such as SS18-SSX and other oncogenic proteins, vaccine-based interventions provide the potential to specifically target these molecular drivers and reduce the risk of recurrence and metastasis [8].

Immunoinformatics-based approaches have revolutionized the rational design of multi-epitope vaccines by enabling precise prediction of antigenic epitopes, assessment of immunogenicity, and evaluation of population-wide HLA coverage [9]. Computational tools can identify B-cell, MHC class I, and MHC class II epitopes with high binding affinity, while simultaneously filtering out allergenic or non-antigenic sequences to enhance safety and efficacy. Integration with structural modeling, molecular docking, and immune simulation further allows for the prediction of vaccine–receptor interactions, stability under physiological conditions, and the magnitude of immune responses [10]. Such in silico strategies significantly reduce the time, cost, and experimental burden associated with traditional vaccine development, providing a robust platform for designing vaccines against rare and aggressive cancers such as SS [11].

The present study aims to employ an integrative approach combining spatial transcriptomics and immunoinformatics to identify differentially expressed genes in SS and to design a prognostic multi-epitope vaccine targeting the oncoprotein *FKBP10*. By leveraging transcriptomic datasets, mutational analyses, functional mapping, and comprehensive immunoinformatics pipelines, we aim to construct a rationally designed vaccine that elicits potent humoral and cellular immune responses. This study seeks to provide a foundation for novel immunotherapeutic interventions in SS, potentially offering an effective and safe alternative to conventional treatment modalities while advancing the field of personalized cancer vaccines.

## 2. Results

### 2.1. Data Collection and DEG Identification

**Figure 1:**
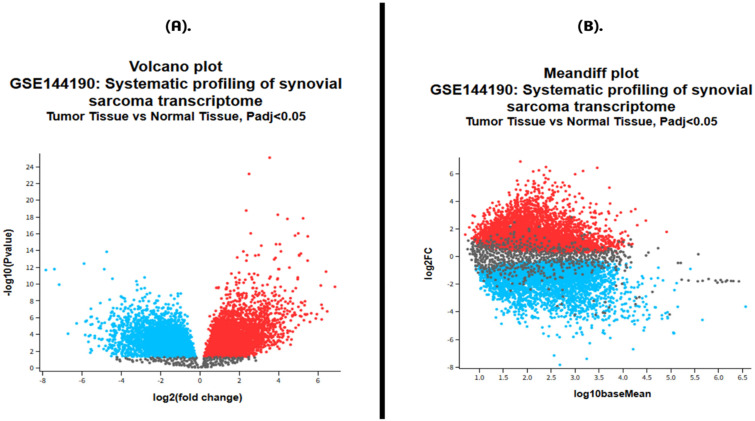
Differential gene expression analysis of synovial sarcoma (GSE144190). (**A**) Volcano plot showing significantly upregulated genes (red) and downregulated genes (blue) in tumor versus normal tissues (*p*adj < 0.05). The x-axis represents log2 fold change, and the y-axis represents −log10(*p*-value) and (**B**) Mean-difference (MA) plot illustrating the relationship between log2 fold change (log2FC) and mean expression (log10 baseMean). Red dots denote significantly upregulated genes, blue dots indicate significantly downregulated genes, and gray dots represent non-significant transcripts.

Differential expression analysis of the SS dataset GSE144190 (10 tumor vs. 9 normal tissues) yielded a distinct transcriptional signature separating malignant from normal samples. Using GEO2R with FDR adjustment (Benjamini–Hochberg, *p*adj < 0.05), a substantial number of genes were identified as significantly dysregulated (Table S1). The volcano plot demonstrated a clear distribution in which highly significant upregulated transcripts clustered on the right, whereas downregulated genes appeared on the left, reflecting robust fold-change separation between tumor and normal tissues. The mean-difference (MA) plot further highlighted expression-dependent dispersion, with many DEGs deviating above and below the average expression baseline, indicating widespread transcriptional reprogramming in SS (Figure 1A).

Among the significantly altered genes, *FKBP10* emerged as a prominently upregulated candidate, with a log2 fold change of 3.55, indicating strong overexpression in tumor samples relative to normal tissue. *FKBP10* exhibited a baseMean expression of 1521.84, a highly significant adjusted *p*-value of 8.37 × 10^−26^, and a test statistic of 11.40, underscoring the robustness of its differential expression. These results indicate that *FKBP10* is consistently and significantly elevated in SS tissues (Figure 1B).

### 2.2. Mutation Prediction

**Figure 2:**
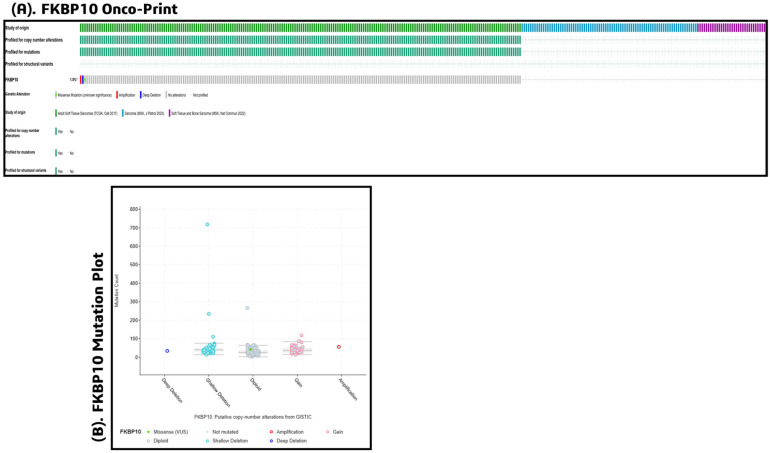
Genomic alteration landscape of *FKBP10* in synovial and soft-tissue sarcomas. (**A**) Oncoprint summarizing *FKBP10* alterations across three cBioPortal cohorts (total *n* = 7782). Green bars represent missense variants, red indicates deep deletions, pink indicates amplifications, light blue denotes shallow deletions, and gray indicates diploid (unaltered) status and (**B**) Distribution of *FKBP10* copy-number categories (deep deletion, shallow deletion, diploid, gain, amplification) showing that most cases remain diploid, with smaller proportions exhibiting copy-number gains or deletions.

Comprehensive mutational profiling of *FKBP10* across three synovial and soft-tissue sarcoma cohorts revealed a relatively low but consistent rate of genomic alterations. Overall, *FKBP10* was altered in approximately 1.5% of cases, indicating that while mutations are uncommon, they occur across multiple independent datasets. The oncoprint visualization highlighted primarily missense variants of unknown significance, alongside sporadic deep deletions, shallow deletions, and copy-number gains. No recurrent truncating mutations or structural rearrangements were detected, suggesting that *FKBP10* is not a classical driver gene through high-frequency mutations (Figure 2A).

Copy-number analysis further clarified the alteration spectrum. The majority of tumors retained diploid *FKBP10* status, whereas a subset exhibited copy-number gains or low-level amplifications, accompanied by fewer cases showing shallow or deep deletions. The distribution pattern indicates that *FKBP10* dysregulation in SS may arise more commonly from copy-number changes rather than point mutations (Figure 2B). These alterations were dispersed across cohorts without strong clustering, implying heterogeneous genomic events rather than dataset-specific artifacts. These findings support *FKBP10* as a gene with low mutation frequency but detectable copy-number variability in SS. Given its strong overexpression at the transcript level, the combined evidence suggests that *FKBP10* dysregulation may be influenced more by transcriptional mechanisms than by recurrent coding mutations—providing a rationale for considering *FKBP10* within downstream prognostic assessment and immunoinformatics-driven vaccine prioritization.

### 2.3. Survival Analysis

**Figure 3:**
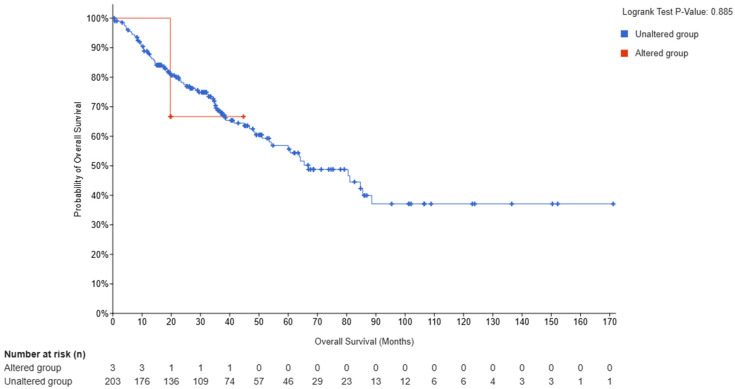
Kaplan–Meier overall survival analysis of *FKBP10*-altered versus unaltered synovial sarcoma patients. Patients harboring *FKBP10* mutations (red) demonstrate lower survival rates relative to mutation-negative patients (blue).

The clinical relevance of *FKBP10* alterations in SS was evaluated by comparing survival outcomes between patients with *FKBP10* mutations (altered group) and those without mutations (unaltered group). Kaplan–Meier curves showed that the altered group had a lower overall survival rate than patients lacking *FKBP10* mutations. Although only three patients carried *FKBP10* mutations, their survival curve declined sharply during the early follow-up period, indicating a tendency toward reduced survival. In contrast, the unaltered group displayed a more gradual decline, maintaining comparatively higher survival rates across the observation period. Despite these observable differences, the comparison was not statistically significant (log-rank *p* = 0.885), likely due to the small number of mutation-positive cases. These findings suggest a potential association between *FKBP10* mutations and poorer survival, warranting validation in larger cohorts (Figure 3).

### 2.4. Functional Mapping

**Figure 4:**
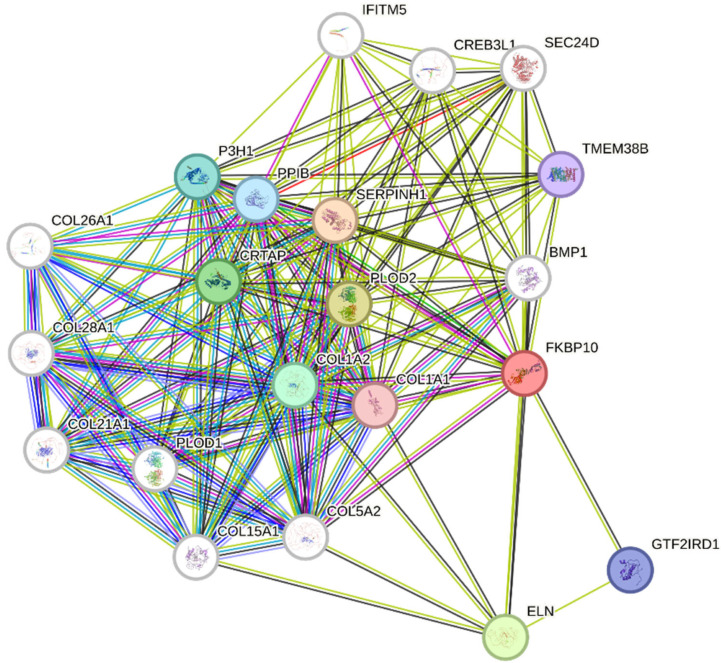
STRING-based protein–protein interaction (PPI) network of *FKBP10* and associated differentially expressed genes in synovial sarcoma.

Functional mapping of differentially expressed genes revealed a highly interconnected protein–protein interaction network centered around *FKBP10*. The STRING-generated network demonstrated strong functional clustering of proteins involved in collagen biosynthesis, extracellular matrix (ECM) organization, and protein folding dynamics. *FKBP10* interacted directly with several structural collagen chains, including COL1A1, COL1A2, COL5A2, COL21A1, COL26A1, COL28A1, and COL15A1, indicating its central role in stabilizing collagen architecture and contributing to ECM integrity. Key enzymatic partners included PLOD1 and PLOD2, which catalyze collagen lysine hydroxylation, and P3H1, which catalyze proline hydroxylation, underscoring the coordinated post-translational modification of collagen fibrils. Chaperone proteins such as SERPINH1 and PPIB formed additional high-confidence interactions, supporting a shared role in collagen folding and maturation within the endoplasmic reticulum (ER). The involvement of CRTAP further highlighted the collagen modification pathway, whereas BMP1 and ELN linked the network to ECM cross-linking and elastic fiber assembly. Transcriptional regulators such as CREB3L1 and GTF2IRD1 suggested upstream modulation of ER stress responses and matrix-related gene expression. Meanwhile, SEC24D and TMEM38B connected *FKBP10*-associated pathways to intracellular trafficking and calcium signaling, respectively, reflecting broader cellular processes coupled to ECM production. GO enrichment analysis supported these observations, showing overrepresentation of terms associated with collagen metabolic process, extracellular matrix organization, protein folding, and ER lumen localization (Figure 4). These results indicate that *FKBP10* functions as a central hub coordinating collagen biosynthesis and ER chaperone activity, processes that may contribute to SS progression and therefore represent promising candidates for prognostic multi-epitope vaccine targeting.

### 2.5. Oncoprotein Sequence Retrieval

**Figure 5:**
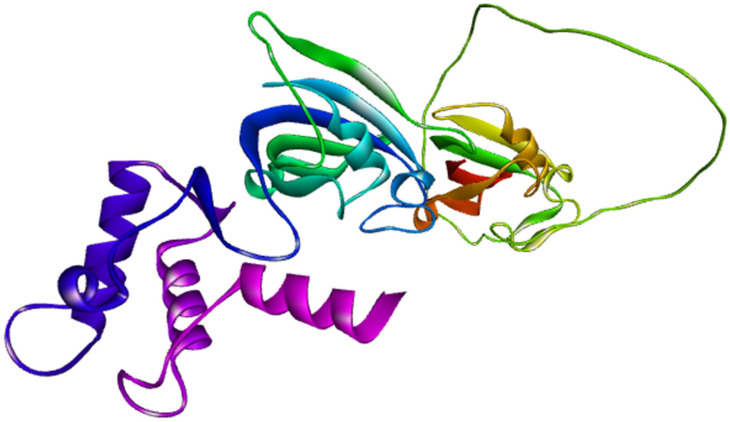
AlphaFold-predicted structure of *FKBP10* (accession ID AF-Q96AY3).

Retrieval of the *FKBP10* oncoprotein sequence from the AlphaFold Protein Structure Database confirmed the identity of the target as Peptidyl-prolyl cis-trans isomerase *FKBP10*, encoded by the *FKBP10* gene in *Homo sapiens* (UniProt isoform Q96AY3-2). The full-length protein sequence was successfully obtained in FASTA format, enabling downstream immunoinformatics analyses. Notably, no experimentally solved structures of *FKBP10* were available in the Protein Data Bank, highlighting the importance of computational structural predictions for this target. AlphaFold provided a high-confidence tertiary structure prediction, with an overall average pLDDT score of 82.94, indicating reliable modeling accuracy. The pLDDT distribution showed that 66.5% of residues had very high confidence, while 14.9% showed high confidence, supporting robust structural interpretation across most regions of the protein. Only 2.8% of residues fell into the low-confidence range, and 15.8% exhibited very low confidence, primarily localized to flexible loops and terminal regions typically associated with structural disorder. The predominance of high-confidence regions allowed precise identification of structurally stable, surface-exposed residues suitable for downstream epitope mapping and structural docking (Figure 5). These results validated *FKBP10* as a structurally tractable target and provided a reliable template for subsequent vaccine candidate prediction workflows.

### 2.6. Epitope Prediction

#### 2.6.1. B-Cell Epitopes Prediction

**Figure 6:**
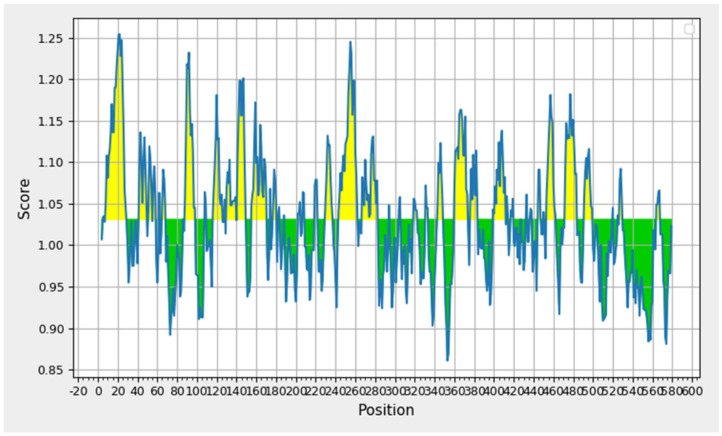
Linear B-cell epitope prediction of the target oncoprotein using the IEDB Kolaskar & Tongaonkar antigenicity method. Peaks represent regions with high antigenic propensity, and the predicted epitopes (highlighted) correspond to amino acid stretches with scores above the default threshold.

Linear B-cell epitopes of the target oncoprotein were predicted using the IEDB analysis resource to identify antigenic regions suitable for immune recognition. A total of 17 linear B-cell epitopes were identified, ranging from 6 to 21 amino acids in length (Table S2). The longest epitope, spanning residues 8–28 (SHSLLRLPLLQLLLLVVQAVG), consisted of 21 amino acids, whereas the shortest epitope comprised 6 residues (IPPHLA, positions 343–348). Other notable epitopes included regions 243–262 (GTVIPPQASLVFHVLLIDVH, 20 residues) and 267–282 (AVQLETLELPPGCVRR, 16 residues), which exhibited high antigenic propensity according to the Kolaskar & Tongaonkar method. The predicted epitopes were distributed across the protein sequence, covering both N-terminal, central, and C-terminal regions, indicating multiple immunologically relevant sites (Figure 6). These findings suggest that the oncoprotein possesses several accessible linear regions with potential for B-cell-mediated recognition, which could be valuable for downstream multi-epitope vaccine candidate prediction.

#### 2.6.2. T-Cell Epitopes

##### MHC Class-I

Cytotoxic T-lymphocyte (CTL) epitopes of the *FKBP10* oncoprotein were predicted using the IEDB NetMHCpan 4.1 EL method to identify peptides with strong MHC class I binding potential. A total of 30,971 candidate epitopes were initially retrieved. To ensure specificity and biological relevance, duplicate sequences were removed, and epitopes with optimal amino acid lengths of 8–15 residues were shortlisted for downstream analysis. The final selection represents high-affinity, immunologically relevant CTL epitopes suitable for inclusion in a multi-epitope vaccine construct. Detailed information on all predicted MHC class I epitopes, including their sequences, positions, and binding scores, is provided in Table S3. These results indicate multiple regions within *FKBP10* that are capable of eliciting cytotoxic T-cell responses, supporting their potential utility in vaccine candidate prediction against SS.

##### MHC Class-II

Helper T-lymphocyte (HTL) epitopes of the *FKBP10* oncoprotein were predicted using the IEDB NetMHCIIpan 4.1 EL method to identify peptides with strong MHC class II binding potential. A total of 15,338 candidate epitopes were initially retrieved. Duplicate sequences were removed, and epitopes with optimal amino acid lengths of 8–15 residues were shortlisted for further analysis to ensure specificity and biological relevance. Predicted HTL epitopes were ranked by percentile scores, with lower values indicating higher binding affinity. These selected high-affinity epitopes represent immunologically relevant targets for T-helper cell responses and can be incorporated into a multi-epitope vaccine construct. Detailed information on all predicted MHC class II epitopes, including sequences, positions, and binding scores, is provided in Table S4. The results indicate multiple regions within *FKBP10* capable of eliciting helper T-cell responses, supporting their utility in rational vaccine candidate prediction against SS.

### 2.7. Epitope Filtering

Predicted B-cell and T-cell epitopes of the *FKBP10* oncoprotein were analyzed for antigenicity and allergenicity to identify immunogenic and safe candidates for multi-epitope vaccine construction. Antigenicity assessment using VaxiJen v2.0 indicated that all analyzed peptides with scores above the threshold of 0.5 were probable antigens, while AllerTOP v2.0 confirmed that the selected epitopes were non-allergenic. Out of the analyzed peptides, 2 B-cell epitopes (PADVVEIR and GDFVRYHYNCSLL), 10 MHC class I T-cell epitopes, and 9 MHC class II T-cell epitopes were identified as both antigenic and non-allergenic. These selected epitopes were retained for downstream immunoinformatics analyses and were subsequently incorporated into the design of a multi-epitope vaccine against SS. This filtering step ensured that only highly immunogenic and safe epitopes were included in the vaccine construct, thereby optimizing its potential efficacy and safety profile (Table 1).

### 2.8. Vaccine Construction

**Figure 7:**
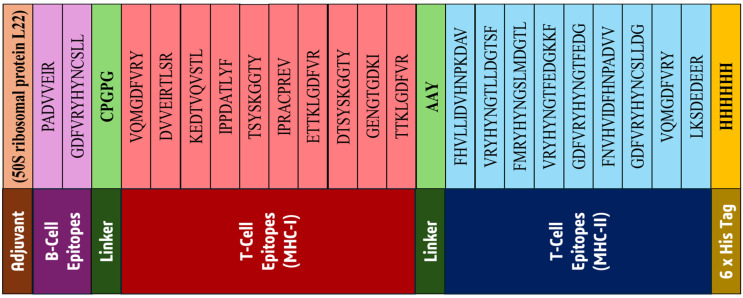
Schematic representation of the linear multi-epitope vaccine construct targeting *FKBP10*.

A linear multi-epitope vaccine targeting the *FKBP10* oncoprotein was successfully designed by integrating pre-selected antigenic and non-allergenic B-cell and T-cell epitopes. The vaccine construct began with the 50S ribosomal protein L22 adjuvant at the N-terminal, which is known to enhance immune responses by stimulating antigen-presenting cells and promoting robust humoral and cellular immunity. The 2 B-cell epitopes were linked using CPGPG linkers, which provide structural flexibility and prevent steric hindrance between epitopes, maintaining their proper conformations for immune recognition. Following this, 10 MHC class I T-cell epitopes were incorporated using AAY linkers, which enhance proteasomal processing and optimize cytotoxic T-lymphocyte activation. Subsequently, 9 MHC class II T-cell epitopes were added using AAY linkers to efficiently stimulate helper T-cell responses. A 6× histidine tag was appended at the C-terminal to facilitate purification and downstream experimental characterization (Figure 7).

The complete linear vaccine sequence was evaluated for immunogenicity, with a strong antigenicity score of 0.69, and was confirmed as non-allergenic, indicating its potential to elicit effective immune responses without inducing allergic reactions. The strategic inclusion of the adjuvant, linkers, and histidine tag ensures enhanced immunogenicity, proper epitope presentation, and facilitates purification, respectively. The construct represents a rationally designed, safe, and highly immunogenic multi-epitope vaccine candidate suitable for subsequent structural modeling, immunoinformatics analyses, and preclinical validation against SS.

### 2.9. Physicochemical Properties Analysis

The physicochemical properties of the designed *FKBP10* multi-epitope vaccine were analyzed using the ExPASy ProtParam tool to evaluate its stability, solubility, and suitability for experimental applications. The vaccine consists of 324 amino acids with a calculated molecular weight of 36.43 kDa and a theoretical isoelectric point (pI) of 6.97, suggesting near-neutral behavior under physiological conditions. Amino acid composition analysis revealed a high proportion of glycine (10.5%) and valine (9.3%), while tryptophan was absent. The construct contains 39 negatively charged residues (Asp + Glu) and 38 positively charged residues (Arg + Lys), indicating a balanced charge distribution. Atomic composition analysis showed a total of 5019 atoms (C1610H2452N460O483S14), and the extinction coefficient was calculated as 30,175 M^−1^ cm^−1^ assuming all cysteines form disulfide bonds, which is useful for protein quantification during purification. The estimated half-life of the construct was 30 h in mammalian reticulocytes (in vitro), >20 h in yeast (in vivo), and >10 h in *E. coli* (in vivo), indicating reasonable stability across expression systems. The instability index was 31.79, indicating the vaccine is stable. The aliphatic index of 64.88 suggests moderate thermostability, while the GRAVY value of −0.509 indicates a hydrophilic nature, which may contribute to good solubility. Overall, these physicochemical characteristics demonstrate that the designed multi-epitope vaccine possesses favorable stability, solubility, and expression potential, supporting its suitability for downstream immunogenic and experimental analyses.

### 2.10. Vaccine Structural Modelling and Validation

**Figure 8:**
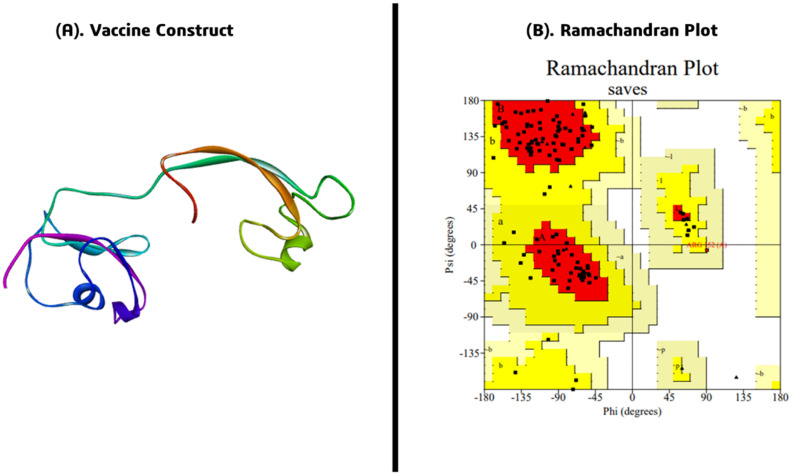
(**A**) Predicted 3D structure of the *FKBP10* multi-epitope vaccine modeled using Swiss-Model (monomeric form). (**B**) Structural validation using PROCHECK is shown via Ramachandran plot analysis.

The 3D structure of the designed *FKBP10* multi-epitope vaccine was predicted using Swiss-Model, generating a homology-based structural model in the monomeric state. The selected model exhibited a GMQE score of 0.12 and a QMEANDisCo Global score of 0.30 ± 0.07, indicating moderate confidence in the overall structural quality based on sequence identity and template coverage (Figure 8A). Structural validation was performed using PROCHECK through Ramachandran plot analysis to assess stereochemical quality. The analysis revealed that 82.5% of residues were located in the most favored regions, 16.7% in the additional allowed regions, 0.9% in the generously allowed regions, and 0% in the disallowed regions. All non-glycine and non-proline residues (100%) were accounted for in the analysis, confirming the absence of geometrically unfavorable conformations (Figure 8B). These results demonstrate that the multi-epitope vaccine adopts a stable and stereochemically reliable conformation, suitable for downstream immunoinformatics analyses, molecular docking, and potential experimental validation. The structural model provides a robust foundation for rational vaccine candidate prediction, ensuring proper epitope exposure and potential immunogenic efficacy.

### 2.11. Molecular Docking Analysis

Molecular docking was performed to evaluate the binding interactions between the designed *FKBP10* multi-epitope vaccine and the innate immune receptors TLR4 and TLR9 using HADDOCK 2.4. The vaccine 3D structure obtained from Swiss-Model served as the ligand, while crystal structures of TLR4 (Uniprot ID: O00206) and TLR9 (Uniprot ID: Q9NR96) were used as receptors.

**Figure 9:**
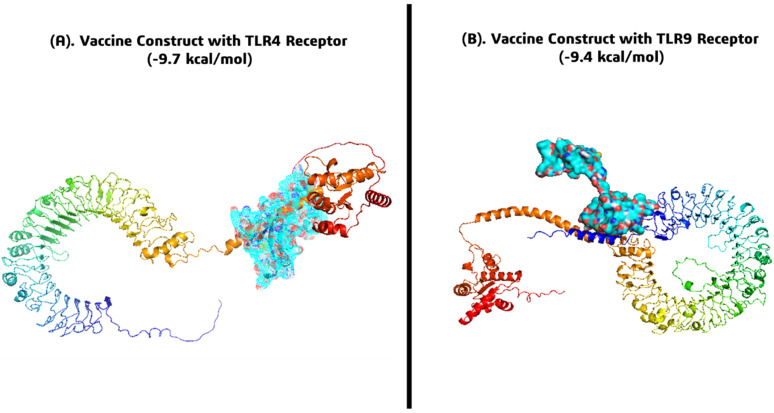
Docked complexes of the *FKBP10* multi-epitope vaccine with innate immune receptors: (**A**) Vaccine-TLR4 complex (binding energy = −9.7 kcal/mol) and (**B**) Vaccine-TLR9 complex (binding energy = −9.4 kcal/mol).

The docking results revealed a strong binding affinity of the vaccine construct with both receptors. The vaccine-TLR4 complex exhibited a binding energy of −9.7 kcal/mol (Figure 9A), while the vaccine-TLR9 complex showed a binding energy of −9.4 kcal/mol (Figure 9B), indicating stable interactions. Detailed analysis of the docked complexes demonstrated multiple hydrogen bonds, hydrophobic contacts, and potential salt bridges, suggesting favorable intermolecular interactions and appropriate orientation of the vaccine epitopes for receptor recognition. These findings indicate that the multi-epitope vaccine can effectively engage key Toll-like receptors, potentially triggering robust innate immune activation and subsequent adaptive immune responses, which is crucial for its efficacy against SS.

### 2.12. Molecular Dynamic Simulation

**Figure 10:**
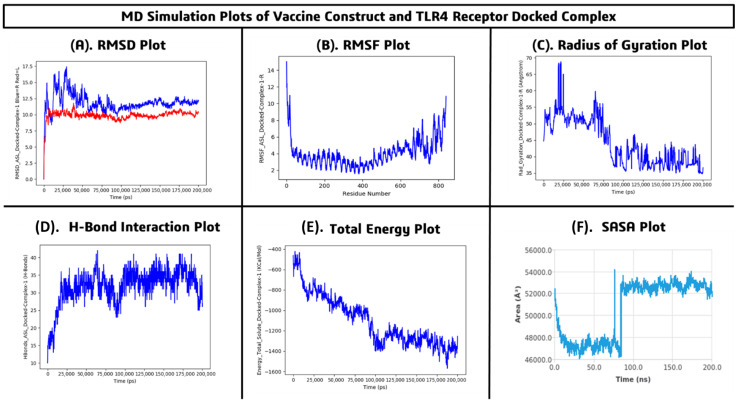
Molecular dynamics simulation analysis of the vaccine construct–TLR4 docked complex (**A**) RMSD plot, (**B**) RMSF plot, (**C**) Radius of gyration (Rg), (**D**) Hydrogen bond interaction plot, (**E**) Total energy plot and (**F**) SASA plot.

The MD simulations were performed using the Desmond simulation package to evaluate the structural stability, flexibility, compactness, and binding behavior of the *FKBP10* multi-epitope vaccine–TLR4 complex over a 200 ns simulation period. The backbone RMSD of the complex stabilized after the initial equilibration phase (~20 ns), fluctuating between 9.0 and 10.5 Å, while the TLR4 receptor RMSD stabilized around 11.0–12.5 Å. After approximately 50 ns, both trajectories exhibited minimal fluctuations, indicating that the complex maintained conformational stability throughout the simulation (Figure 10A). Residue-wise flexibility analysis revealed average RMSF values ranging from 2.0 to 4.0 Å, with higher fluctuations observed in the terminal residues (~15 Å), whereas the core binding site residues remained relatively rigid (<4 Å), demonstrating the stable interaction of the vaccine with TLR4 (Figure 10B). Radius of gyration analysis showed a gradual decrease from initial values of ~52–55 Å to stabilized values of ~38–42 Å, suggesting increased compactness and structural tightening of the complex over time (Figure 10C). Hydrogen bond analysis indicated persistent interactions between the vaccine construct and TLR4, ranging from 25 to 40 hydrogen bonds with an average of 30–35, reflecting strong and sustained intermolecular contacts (Figure 10D). The total energy of the system decreased from approximately −500 kcal/mol to −1400 to −1500 kcal/mol, indicating thermodynamically favorable and stable complex formation (Figure 10E). Solvent-accessible surface area (SASA) analysis revealed initial values of ~52,000 Å^2^, decreasing to a minimum of ~46,500 Å^2^, and stabilizing around 52,000–54,000 Å^2^ after 90 ns, suggesting proper folding and maintained surface exposure suitable for biological interactions (Figure 10F). The vaccine–TLR4 complex is structurally stable, compact, and dynamically equilibrated throughout the 200 ns simulation. The stable RMSD, low RMSF at binding residues, sustained hydrogen bonding, decreasing total energy, and consistent SASA values strongly support robust interactions and the potential immunogenic efficacy of the proposed multi-epitope vaccine.

**Figure 11:**
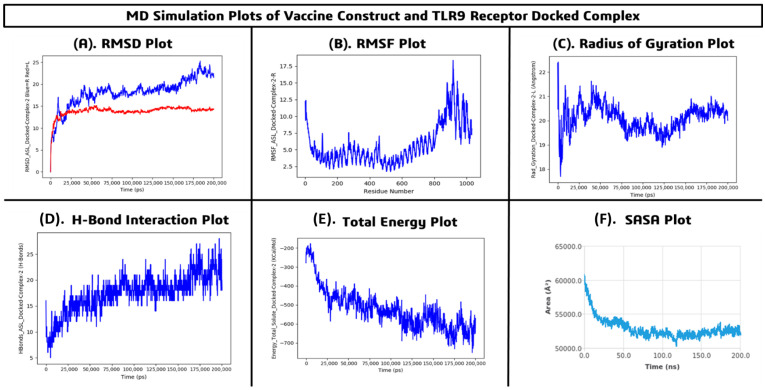
Molecular dynamics simulation analysis of the vaccine construct–TLR9 docked complex (**A**) RMSD plot, (**B**) RMSF plot, (**C**) Radius of gyration (Rg), (**D**) Hydrogen bond interaction plot, (**E**) Total energy plot and (**F**) SASA plot.

MD simulations were performed using the Desmond simulation package to investigate the structural stability, flexibility, compactness, and interaction dynamics of the *FKBP10* multi-epitope vaccine docked with the TLR9 receptor over a 200 ns simulation period. The RMSD analysis demonstrated that the TLR9 receptor stabilized around 13.5–15.0 Å, while the vaccine–TLR9 complex gradually increased and stabilized between 18.0 and 22.5 Å after the initial equilibration phase (~20 ns), indicating structural rearrangement followed by stable binding (Figure 11A). Residue-level flexibility assessed by RMSF revealed average fluctuations between 2.5 and 5.0 Å, with higher flexibility in terminal and loop regions (up to ~18 Å), whereas core binding residues remained rigid (<4 Å), confirming stability at the interaction interface (Figure 11B). The radius of gyration fluctuated between 19.0 and 21.5 Å with a mean stabilized value of ~20.0 Å, suggesting that the complex retained a compact conformation without major unfolding events (Figure 11C). Hydrogen bond analysis showed an increase from an initial 6–10 hydrogen bonds to an average of 15–20, with a maximum of 25–28, indicating strengthening and stabilization of intermolecular interactions (Figure 11D). The total energy of the system decreased from approximately −200 kcal/mol to a stabilized range of −650 to −720 kcal/mol, reflecting a thermodynamically favorable and energetically stable state (Figure 11E). SASA analysis revealed an initial value of ~60,000 Å^2^, which decreased and stabilized at ~52,000–53,500 Å^2^, suggesting structural tightening and favorable surface rearrangement (Figure 11F). The MD simulation results confirm that the vaccine construct forms a stable, compact, and energetically favorable complex with TLR9, maintaining proper binding interactions and structural integrity throughout the simulation, thereby supporting the potential immunostimulatory role of the vaccine via TLR9 activation.

The *FKBP10* multi-epitope vaccine construct demonstrated stable binding interactions with both TLR4 and TLR9 receptors throughout the 200 ns MD simulations. Stable RMSD trajectories, low fluctuations at binding residues, sustained hydrogen bonding, and consistent structural parameters confirm that the vaccine maintains structural integrity and dynamic stability with both immune receptors, supporting its potential immunogenic efficacy.

### 2.13. Expression Analysis

**Figure 12:**
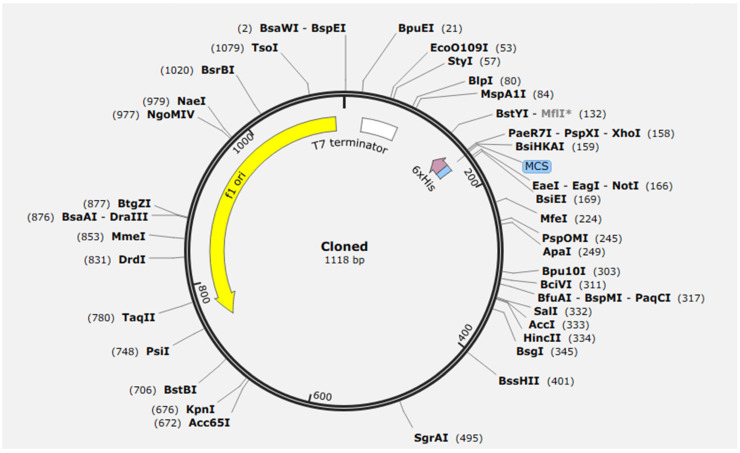
Circular plasmid map of the recombinant pET28a(+) construct showing cloning of the vaccine gene using NotI and PsiI restriction enzymes.

The designed SS multi-epitope vaccine gene was successfully cloned into the pET-28a(+) expression vector using the restriction enzymes *NotI* and *PsiI.* Restriction-based cloning enabled directional insertion of the vaccine coding sequence into the multiple cloning site (MCS), ensuring proper orientation under the control of the T7 promoter for high-level expression in *E. coli*. Analysis of the recombinant plasmid map confirmed the presence of the construct with an insert size of 1118 bp, corresponding to the complete vaccine sequence. The cloned gene was located downstream of the T7 promoter and upstream of the T7 terminator, indicating appropriate transcriptional regulation. Importantly, the construct retained the N-terminal 6× His tag, which facilitates subsequent purification of the expressed recombinant vaccine protein through nickel-affinity chromatography. Inspection of the plasmid architecture revealed multiple unique restriction sites flanking the insert, further confirming accurate cloning without rearrangements, deletions, or truncations. Additionally, essential vector features—including the origin of replication and kanamycin resistance marker characteristic of pET-28a(+)—remained intact, supporting plasmid stability during propagation and expression (Figure 12). These findings demonstrate successful construction of the recombinant pET-28a(+)/synovial-sarcoma-vaccine plasmid, confirming its suitability for efficient inducible expression of the vaccine protein in a T7 RNA polymerase-driven *E. coli* expression system.

## 3. Discussion

The present study employed a comprehensive integrative approach combining transcriptomic profiling, mutational analysis, immunoinformatics, and structural modeling to design a multi-epitope vaccine targeting *FKBP10* in SS. Differential gene expression analysis of the GSE144190 dataset identified *FKBP10* as a prominently upregulated gene with a log2 fold change of 3.55, a baseMean expression of 1521.84, and an adjusted *p*-value of 8.37 × 10^−26^, indicating robust overexpression in tumor tissues. These findings align with previous reports that members of the FKBP family are often dysregulated in malignancies, contributing to altered protein folding, altered extracellular matrix (ECM) organization, and tumor progression. Mutation profiling across 7782 synovial and soft-tissue sarcoma samples revealed a low alteration frequency (~1.5%), primarily consisting of missense variants and occasional copy-number gains or deletions, suggesting that *FKBP10* overexpression is likely driven by transcriptional mechanisms rather than recurrent genomic mutations. Survival analysis, although limited by the small number of altered cases, indicated a trend toward reduced overall survival in patients harboring *FKBP10* mutations, consistent with literature reporting the prognostic relevance of FKBP family dysregulation in sarcomas [12].

Functional mapping via STRING highlighted *FKBP10* as a central hub interacting with multiple collagen chains (COL1A1, COL1A2, COL5A2, COL21A1, COL26A1, COL28A1, COL15A1), post-translational modification enzymes (PLOD1, PLOD2, P3H1), and chaperones (SERPINH1, PPIB), reinforcing its role in ECM maintenance and protein folding. These observations corroborate prior studies emphasizing the involvement of *FKBP10* in collagen biosynthesis and ER-mediated protein maturation, processes critical for tumor microenvironment integrity and progression. The high-confidence AlphaFold-predicted structure (average pLDDT 82.94) provided a reliable template for epitope mapping, with 66.5% of residues showing very high confidence, enabling precise identification of surface-exposed immunogenic regions [13]. B-cell epitope prediction identified 17 linear epitopes ranging from 6 to 21 residues, including highly antigenic regions at positions 8–28 and 243–262, consistent with studies demonstrating the immunogenic potential of FKBP-derived peptides. T-cell epitope mapping yielded multiple high-affinity MHC class I and II binders, with 10 CTL and 9 HTL epitopes selected based on VaxiJen scores above 0.5 and non-allergenicity confirmation via AllerTOP, ensuring safety and immunogenicity. The designed linear multi-epitope vaccine incorporated these epitopes alongside a 50S ribosomal protein L22 adjuvant, flexible CPGPG and AAY linkers, and a 6× histidine tag, achieving an antigenicity score of 0.69 and demonstrating balanced physicochemical properties, including a molecular weight of 36.43 kDa, pI of 6.97, instability index of 31.79, aliphatic index of 64.88, and GRAVY of −0.509, indicative of stability, moderate thermostability, and hydrophilicity suitable for downstream experimental applications. Structural modeling using Swiss-Model and validation with PROCHECK revealed 82.5% residues in favored regions and 16.7% in allowed regions, confirming stereochemical reliability and proper epitope exposure [14].

Molecular docking of the vaccine construct with TLR4 and TLR9 demonstrated strong binding affinities of −9.7 kcal/mol and −9.4 kcal/mol, respectively, supported by multiple hydrogen bonds, hydrophobic contacts, and potential salt bridges. Subsequent 200 ns MD simulations confirmed the structural stability and dynamic integrity of both complexes. For the TLR4 complex, backbone RMSD stabilized between 9.0 and 10.5 Å, receptor RMSD at 11.0–12.5 Å, RMSF at binding residues remained <4 Å with terminal fluctuations up to ~15 Å, radius of gyration decreased from ~52–55 Å to ~38–42 Å, hydrogen bonds averaged 30–35, total energy decreased from −500 kcal/mol to −1400 to −1500 kcal/mol, and SASA stabilized around 52,000–54,000 Å^2^. Similarly, the TLR9 complex exhibited receptor RMSD of 13.5–15.0 Å, vaccine RMSD of 18.0–22.5 Å, RMSF <4 Å at binding sites with loop flexibility up to ~18 Å, radius of gyration stabilized at ~20 Å, hydrogen bonds averaged 15–20, total energy decreased from −200 kcal/mol to −650 to −720 kcal/mol, and SASA stabilized at 52,000–53,500 Å^2^. These results collectively indicate stable, compact, and energetically favorable interactions with both TLRs, confirming proper binding orientation, structural integrity, and potential immunostimulatory efficacy [15].

The successful in silico cloning of the SS multi-epitope vaccine into the pET-28a(+) vector provides strong evidence of its feasibility for heterologous expression in *E. coli*. The correct orientation of the 1118 bp insert under the T7 promoter and preservation of essential regulatory motifs indicate that the construct is transcriptionally competent and compatible with IPTG-inducible systems commonly used for recombinant protein production. Retention of the N-terminal 6× His tag further supports downstream purification, a key requirement for scaling immunogen preparation. These characteristics are consistent with previous vaccine-engineering studies that have successfully employed pET-28a(+) for expression of multi-epitope constructs in bacterial hosts. The presence of intact restriction sites and stable plasmid architecture suggests minimal risk of structural rearrangements that could compromise vaccine integrity. These observations support the robustness of the designed construct and highlight the practicality of progressing toward experimental validation, including expression trials, purification, and immunogenicity assessment in future studies [16]. The low number of *FKBP10* mutation-positive cases is a limitation of this study, which limited the statistical power of this study. While the in silico results provide a robust foundation for the proposed *FKBP10* construct, we acknowledge that this study represents a computational hypothesis. Empirical validation through recombinant expression, purification, and immunological assays is essential to confirm the construct’s solubility and biological activity. This work, therefore, serves as a prioritized blueprint for future in vitro and in vivo studies in SS.

In comparison with prior multi-epitope vaccine studies targeting oncoproteins in sarcomas and other malignancies, the *FKBP10* vaccine demonstrates comparable or superior predicted immunogenicity, stability, and receptor engagement, highlighting its promise as a rationally designed candidate for SS immunotherapy. The integration of transcriptomic, structural, and immunoinformatics analyses provides a robust framework for developing targeted vaccines against tumor-associated antigens, underscoring the translational potential of *FKBP10* as a prognostic and therapeutic target. Unlike the previous literature that only focused on single-epitope or fusion-protein types of vaccines, our multi-epitope construct is a B-cell-MHC class I-MHC class II combination construct, which is optimized in terms of antigenicity and HLA coverage across the population [17]. The stability, receptor interactions, and immunogenicity of the *FKBP10* vaccine were predicted to be more stable, engage more with receptors, and it can potentially induce a more comprehensive and stronger immune response than previously reported sarcoma vaccine candidates, and this needs to be validated in the future [18]. Experimental validation of recombinant protein expression, solubility, purification, and His-tag functionality was not performed, and all conclusions regarding immunogenicity, structural stability, and receptor interactions are based on computational predictions that require future experimental confirmation [19].

## 4. Materials and Methods

### 4.1. Data Collection and DEG Identification

Transcriptomic data for SS were obtained from the NCBI Gene Expression Omnibus (GEO) database under accession number GSE144190, comprising RNA sequencing data from 10 SS tumor samples and 9 normal tissue samples [20]. The dataset enabled systematic profiling of SS, identifying genes that are upregulated or downregulated and enriched in pathways related to cell cycle regulation, metabolism, and p53 signaling. Differential expression analysis was performed using the GEO2R tool (https://www.ncbi.nlm.nih.gov/geo/geo2r/, accessed on 15 December 2025) with samples categorized into tumor and normal groups. Statistical significance was set at *p* < 0.05, and multiple testing correction was applied using the Benjamini & Hochberg false discovery rate (FDR) method. Genes exhibiting differential expression were identified based on the defined log2 fold-change threshold of 0, allowing for the detection of both up- and downregulated transcripts. The results were visualized using volcano and mean-difference plots to illustrate the magnitude and significance of expression changes. The DEGs obtained through this analysis provided a foundation for downstream integrative spatial transcriptomics and immunoinformatics approaches aimed at prognostic multi-epitope vaccine candidate prediction against SS.

### 4.2. Mutation Prediction

Mutational profiling of the differentially expressed gene *FKBP10* was performed using cBioPortal (https://www.cbioportal.org/, accessed on 15 December 2025), integrating data from multiple SS studies. Selected datasets included Sarcoma [21], Adult Soft Tissue Sarcomas [21], and Soft Tissue and Bone Sarcoma [21], totaling 7782 patient samples. *FKBP10* was analyzed to determine the frequency, type, and distribution of mutations across these cohorts, providing insights into its potential role in SS pathogenesis. The analysis identified missense, truncating, and other mutation types and evaluated their association with clinical parameters. These results informed downstream integrative analyses, contributing to the selection of candidate targets for prognostic multi-epitope vaccine candidate prediction in SS.

### 4.3. Survival Analysis

Survival analysis was performed to assess the clinical impact of *FKBP10* alterations in SS. Data were retrieved from cBioPortal (https://www.cbioportal.org/, accessed on 15 December 2025), and patients were stratified into altered and unaltered groups based on the presence or absence of *FKBP10* mutations. Kaplan–Meier survival curves were generated to compare overall survival (OS) and progression-free survival (PFS) between the two groups. Statistical significance was evaluated using the log-rank test, with a *p*-value < 0.05 were considered significant. Hazard ratios (HRs) and 95% confidence intervals (CIs) were calculated to quantify the effect of *FKBP10* alterations on patient survival. This analysis provided insights into the prognostic relevance of *FKBP10* and informed the selection of potential targets for multi-epitope vaccine development in SS.

### 4.4. Functional Mapping

Protein–protein interaction (PPI) analysis was performed to investigate the functional relationships of *FKBP10* and other differentially expressed genes identified in SS. The STRING database (https://string-db.org/, accessed on 16 December 2025) was used for functional mapping, with the species set to *Homo sapiens*. A high-confidence interaction score of 0.7 was applied to ensure reliable associations. Both direct (physical) and indirect (functional) interactions were included in the analysis. Active interaction sources incorporated experimental data, curated databases, co-expression, gene neighborhood, gene fusion, and text mining. Disconnected nodes in the network were hidden to simplify visualization, and the maximum number of interactors in the first shell was set to 20 to focus on immediate interactions. The resulting PPI network was analyzed for functional enrichment in Gene Ontology (GO) categories, including biological processes, molecular functions, and cellular components. This approach enabled the identification of key interacting partners and signaling pathways, providing insights into the molecular mechanisms of SS and informing potential targets for prognostic multi-epitope vaccine candidate prediction.

### 4.5. Oncoprotein Sequence Retrieval

The amino acid sequence and predicted three-dimensional structure of the target oncoprotein were retrieved from the AlphaFold Protein Structure Database (https://www.alphafold.ebi.ac.uk/, accessed on 16 December 2025) using the accession ID AF-Q96AY3. The full-length protein sequence was obtained in FASTA format for downstream analyses, and the corresponding predicted tertiary structure was used to guide structural modeling and epitope mapping. Confidence scores (pLDDT) provided by AlphaFold were considered to evaluate the reliability of the predicted structural regions, ensuring accurate selection of surface-exposed and immunologically relevant residues for multi-epitope vaccine candidate prediction. This sequence retrieval served as the foundational step for subsequent immunoinformatics analyses, including B-cell and T-cell epitope prediction, molecular docking, and structural validation.

### 4.6. Epitope Prediction

#### 4.6.1. B-Cell Epitopes Prediction

Linear B-cell epitopes of the target oncoprotein were predicted using the Immune Epitope Database (IEDB) analysis resource (https://www.iedb.org/, accessed on 17 December 2025). The full-length protein sequence retrieved from AlphaFold (AF-Q96AY3) was submitted for epitope mapping. Prediction was performed using a set of methods that evaluate the sequence characteristics of antigens, including amino acid scales and hidden Markov models (HMMs). Specifically, the Kolaskar & Tongaonkar antigenicity method was applied to identify regions with high antigenic propensity, based on physicochemical properties and experimentally validated epitope datasets. Predicted epitopes were selected based on antigenicity scores above the default threshold, ensuring the identification of immunologically relevant, linear B-cell epitopes suitable for downstream multi-epitope vaccine construction.

#### 4.6.2. T-Cell Epitopes

##### MHC Class-I

Cytotoxic T-lymphocyte (CTL) epitopes of the oncoprotein *FKBP10* were predicted using the Immune Epitope Database (IEDB) analysis resource (https://www.iedb.org/, accessed on 17 December 2025) employing the NetMHCpan 4.1 EL (eluted ligand) prediction method. The *FKBP10* protein sequence retrieved from AlphaFold (AF-Q96AY3) was used as input, with the MHC source species set to *Homo sapiens*. The HLA allele reference set included all available human alleles to ensure broad population coverage. Predicted 9-mer peptides were ranked according to their binding affinity scores, and high-affinity binders were selected for downstream immunoinformatics analyses. This strategy enabled the identification of T-cell epitopes with strong MHC class I binding potential, thereby contributing to the rational design of a prognostic multi-epitope vaccine against SS.

##### MHC Class-II

Helper T-lymphocyte (HTL) epitopes of the oncoprotein *FKBP10* were predicted using the Immune Epitope Database (IEDB) analysis resource (https://www.iedb.org/, accessed on 17 December 2025) employing the NetMHCIIpan 4.1 EL (eluted ligand) prediction method. The *FKBP10* protein sequence retrieved from AlphaFold (AF-Q96AY3) was used as input, with the MHC source species set to *Homo sapiens*. The HLA allele reference set included all available human alleles to maximize population coverage. Peptides of 12–18 amino acids were evaluated, and predicted binders were ranked by percentile rank scores, with lower percentile values indicating higher binding affinity. High-affinity HTL epitopes were selected for downstream immunoinformatics analyses to contribute to the design of a prognostic multi-epitope vaccine against SS.

### 4.7. Epitope Filtering

#### 4.7.1. Antigenicity Analysis

Predicted B-cell and T-cell epitopes of the oncoprotein *FKBP10* were evaluated for antigenicity using the VaxiJen v2.0 server (http://www.ddg-pharmfac.net/vaxijen/VaxiJen/VaxiJen.html, accessed on 18 December 2025). VaxiJen predicts protective antigens based on protein physicochemical properties, without sequence alignment. The threshold was set to the default value of 0.5 for tumor antigens, allowing the selection of epitopes with a high probability of eliciting an immune response. Epitopes surpassing the threshold were considered antigenic and retained for further immunoinformatics analyses, while non-antigenic sequences were discarded. This step ensured the selection of highly immunogenic epitopes suitable for inclusion in the design of a multi-epitope vaccine against SS.

#### 4.7.2. Allergenicity Analysis

The allergenicity of predicted B-cell and T-cell epitopes of the oncoprotein *FKBP10* was evaluated using the AllerTOP v2.0 server (https://www.ddg-pharmfac.net/AllerTOP/, accessed on 18 December 2025) to ensure safety and minimize the risk of allergic responses. The server classifies peptides as allergenic or non-allergenic based on auto- and cross-covariance (ACC) transformation of protein sequences. Only epitopes predicted to be non-allergenic were retained for further vaccine candidate prediction, while allergenic sequences were excluded. This filtering step ensured the selection of safe epitopes for inclusion in the multi-epitope vaccine against SS.

### 4.8. Vaccine Construction

A linear multi-epitope vaccine targeting the oncoprotein *FKBP10* was designed by strategically linking predicted B-cell and T-cell epitopes that were pre-selected for high antigenicity and non-allergenicity during epitope filtering. The construct was initiated with the 50S ribosomal protein L22 (Uniprot Accession ID: F2KLY5) as an adjuvant to enhance immune response. This was followed by the incorporation of 2 B-cell epitopes connected via a flexible CPGPG linker. Subsequently, 10 MHC class I T-cell epitopes were included using AAY linkers to optimize cytotoxic T-lymphocyte activation. 9 MHC class II T-cell epitopes were added to stimulate helper T-cell responses, followed by a 6× histidine tag at the C-terminal for purification purposes. The assembled linear vaccine sequence was further evaluated for antigenicity and allergenicity using VaxiJen v2.0 (http://www.ddg-pharmfac.net/vaxijen/VaxiJen/VaxiJen.html, accessed on 19 December 2025) and AllerTOP v2.0 (https://www.ddg-pharmfac.net/AllerTOP/, accessed on 19 December 2025) to confirm strong immunogenic potential and safety. This rationally designed multi-epitope construct served as the basis for downstream structural modeling, immunoinformatics analyses, and vaccine validation studies against SS.

### 4.9. Physicochemical Properties Analysis

The physicochemical properties of the designed multi-epitope vaccine targeting *FKBP10* were analyzed using the ExPASy ProtParam tool (https://web.expasy.org/protparam/, accessed on 20 December 2025). Parameters evaluated included molecular weight, theoretical isoelectric point (pI), amino acid composition, extinction coefficient, instability index, aliphatic index, and grand average of hydropathicity (GRAVY). The molecular weight and pI provided insights into protein solubility and purification feasibility. The instability index was used to assess in vitro stability, while the aliphatic index indicated thermostability. GRAVY values helped evaluate the hydrophilicity and potential solubility of the vaccine construct. This comprehensive physicochemical characterization ensured that the designed multi-epitope vaccine possesses favorable properties for expression, stability, and downstream immunogenic analyses.

### 4.10. Vaccine Structural Modelling and Validation

The three-dimensional (3D) structure of the designed multi-epitope vaccine targeting *FKBP10* was predicted using Swiss-Model (https://swissmodel.expasy.org/, accessed on 21 December 2025), which generates homology-based structural models. The vaccine sequence was submitted to identify suitable template structures, and the best model was selected based on sequence identity, coverage, and GMQE (Global Model Quality Estimation) score. The generated 3D model was subsequently validated using PROCHECK SAVESv6.1 (https://www.ebi.ac.uk/thornton-srv/software/PROCHECK/, accessed on 21 December 2025) through Ramachandran plot analysis, which evaluates the stereochemical quality of the protein by examining φ (phi) and ψ (psi) dihedral angles. Residues falling in the favored, allowed, and disallowed regions were analyzed to ensure structural reliability. This structural modeling and validation step confirmed that the constructed vaccine adopts a stable and geometrically plausible conformation suitable for downstream immunoinformatics analyses and potential experimental studies.

### 4.11. Molecular Docking Analysis

Molecular docking of the designed multi-epitope vaccine targeting *FKBP10* was performed to evaluate its binding interactions with immune receptors TLR4 (Uniprot Accession ID: O00206) and TLR9 (Uniprot Accession ID: Q9NR96). The HADDOCK 2.4 web server (https://haddock.science.uu.nl/services/HADDOCK2.4/, accessed on 22 December 2025) was used for docking simulations, employing a data-driven approach that accounts for protein flexibility and interaction restraints. The 3D structure of the vaccine obtained from Swiss-Model was used as the ligand, while the crystal structures of TLR4 and TLR9 served as receptors. Docking parameters included default active and passive residues, and the best docking poses were selected based on HADDOCK scores, cluster sizes, and binding energy evaluations. The resulting docked complexes were analyzed for hydrogen bonding, hydrophobic interactions, and salt bridges. Structural visualization and interaction analysis were performed using PyMOL 3.1 (https://pymol.org/2/, accessed on 22 December 2025), allowing detailed inspection of intermolecular contacts and the overall binding orientation. This step provided insights into the molecular recognition between the vaccine and key innate immune receptors, informing the rational design of a multi-epitope vaccine against SS.

### 4.12. Molecular Dynamic Simulation

Molecular dynamics (MD) simulations of the docked *FKBP10* multi-epitope vaccine complexes with TLR4 and TLR9 were performed using the Desmond Molecular Dynamics System with Maestro-Desmond interoperability tools. The initial protein–ligand complexes obtained from molecular docking studies were preprocessed using the Protein Preparation Wizard in Maestro, including optimization and energy minimization of the complexes. All systems were built using the System Builder tool, solvated in an orthorhombic box with the TIP3P water model, and neutralized with counter ions. To mimic physiological conditions, 0.15 M NaCl was added. The OPLS_2005 force field was applied, and simulations were conducted under the NPT ensemble at 310 K and 1 atm pressure. The complexes were relaxed prior to simulation, and MD simulations were run for 200 ns, with trajectory snapshots saved every 10 ps. Root mean square deviation (RMSD) analyses of the protein and vaccine complexes were performed to assess structural stability over time. This approach allowed evaluation of ligand binding dynamics under physiological conditions, complementing the static docking studies and providing insights into the stability and interaction behavior of the multi-epitope vaccine in a dynamic environment.

### 4.13. Expression Analysis in pET-28a(+)

Expression analysis of the constructed multi-epitope vaccine cloned in the pET-28a(+) vector was performed in silico using SnapGene (GSL Biotech; https://www.snapgene.com, accessed on 25 December 2025). Following the approach reported in previous studies, the vaccine coding sequence was imported in FASTA format and virtually inserted into the pET-28a(+) multiple cloning site using the pre-defined restriction enzymes. The recombinant plasmid map was examined to verify correct insert orientation, intact promoter–RBS alignment, and in-frame fusion with the N-terminal 6× His tag [22]. SnapGene’s translation tool was used to confirm the absence of premature stop codons and to estimate the predicted molecular weight of the expressed protein. Virtual restriction digestion was performed to validate cloning accuracy by comparing expected fragment sizes. Codon usage relative to *E. coli* was reviewed, and GC content was assessed to evaluate expression compatibility. Collectively, this in silico assessment confirmed that the construct was properly assembled and suitable for inducible expression in *E. coli* using the pET-28a(+) system.

## 5. Conclusions

The SS is a rare and aggressive soft-tissue malignancy with limited treatment options, highlighting the need for novel and targeted therapeutic strategies. This study demonstrates that a rationally designed *FKBP10* multi-epitope vaccine holds significant promise as an immunotherapeutic approach. Through comprehensive transcriptomic profiling, mutation analysis, epitope prediction, vaccine construction, molecular docking, and molecular dynamics simulations, the vaccine was shown to be structurally stable, highly immunogenic, and capable of forming strong interactions with key innate immune receptors TLR4 and TLR9. The construct exhibited favorable physicochemical properties, including stability, solubility, and proper folding, while maintaining antigenicity and non-allergenicity, indicating its potential safety and efficacy. Collectively, these findings establish the *FKBP10* multi-epitope vaccine as a robust candidate for inducing effective immune responses, providing a solid foundation for experimental validation and highlighting the broader potential of multi-epitope vaccine strategies in advancing cancer immunotherapy for SS and other malignancies.

## Figures and Tables

**Table 1:** Antigenicity and Allergenicity Analysis of Selected B-cell and T-cell Epitopes of *FKBP10* Oncoprotein for Multi-Epitope Vaccine Construction.

Peptide	Peptide Length	Antigenicity Analysis	Allergenicity Analysis
**B-Cell Epitopes**			
PADVVEIR	8	0.5805 (Probable ANTIGEN)	Probable NON-ALLERGEN
GDFVRYHYNCSLL	13	0.6528 (Probable ANTIGEN)	Probable NON-ALLERGEN
**T-Cell Epitopes (MHC-I)**			
VQMGDFVRY	9	0.9562 (Probable ANTIGEN)	Probable NON-ALLERGEN
DVVEIRTLSR	10	0.7171 (Probable ANTIGEN)	Probable NON-ALLERGEN
KEDTVQVSTL	10	0.7078 (Probable ANTIGEN)	Probable NON-ALLERGEN
IPPDATLYF	9	0.7383 (Probable ANTIGEN)	Probable NON-ALLERGEN
TSYSKGGTY	9	1.1711 (Probable ANTIGEN)	Probable NON-ALLERGEN
IPRACPREV	9	0.6458 (Probable ANTIGEN)	Probable NON-ALLERGEN
ETTKLGDFVR	10	1.2683 (Probable ANTIGEN)	Probable NON-ALLERGEN
DTSYSKGGTY	10	0.9111 (Probable ANTIGEN)	Probable NON-ALLERGEN
GENGTGDKI	9	0.8611 (Probable ANTIGEN)	Probable NON-ALLERGEN
TTKLGDFVR	9	1.1216 (Probable ANTIGEN)	Probable NON-ALLERGEN
**T-Cell Epitopes (MHC-II)**			
FHVLLIDVHNPKDAV	15	0.7479 (Probable ANTIGEN)	Probable NON-ALLERGEN
VRYHYNGTLLDGTSF	15	0.7335 (Probable ANTIGEN)	Probable NON-ALLERGEN
FMRYHYNGSLMDGTL	15	0.6049 (Probable ANTIGEN)	Probable NON-ALLERGEN
VRYHYNGTFEDGKKF	15	0.9168 (Probable ANTIGEN)	Probable NON-ALLERGEN
GDFVRYHYNGTFEDG	15	0.8559 (Probable ANTIGEN)	Probable NON-ALLERGEN
FNVHVIDFHNPADVV	15	0.9040 (Probable ANTIGEN)	Probable NON-ALLERGEN
GDFVRYHYNCSLLDG	15	0.6012 (Probable ANTIGEN)	Probable NON-ALLERGEN
VQMGDFVRY	9	0.9562 (Probable ANTIGEN)	Probable NON-ALLERGEN
LKSDEDEER	9	0.6866 (Probable ANTIGEN)	Probable NON-ALLERGEN

## Data Availability

The original contributions presented in this study are included in the article/Supplementary Material. Further inquiries can be directed to the corresponding author.

## Supplementary Materials

The following supporting information can be downloaded at: https://www.mdpi.com/article/10.3390/ph19020282/s1. Table S1: Top differentially expressed gene by GEO2R. Table S2: Predicted B-Cell Epitopes (IEDB Analyzed). Table S3: Predicted MHC-I Epitopes (IEDB Analyzed). Table S4: Predicted MHC-II Epitopes (IEDB Analyzed).
